# Replication and virulence in pigs of the first African swine fever virus isolated in China

**DOI:** 10.1080/22221751.2019.1590128

**Published:** 2019-03-22

**Authors:** Dongming Zhao, Renqiang Liu, Xianfeng Zhang, Fang Li, Jingfei Wang, Jiwen Zhang, Xing Liu, Lulu Wang, Jiaoer Zhang, Xinzhou Wu, Yuntao Guan, Weiye Chen, Xijun Wang, Xijun He, Zhigao Bu

**Affiliations:** a State Key Laboratory of Veterinary Biotechnology, Harbin Veterinary Research Institute, Chinese Academy of Agricultural Sciences, Harbin, People’s Republic of China; b National High Containment Laboratory for Animal Diseases Control and Prevention, Harbin, People’s Republic of China

**Keywords:** African swine fever virus, virus isolation, animal study, pig, transmission

## Abstract

African swine fever (ASF) entered China in August 2018 and rapidly spread across the entire country, severely threatening the Chinese domestic pig population, which accounts for more than 50% of the pig population worldwide. In this study, an ASFV isolate, Pig/Heilongjiang/2018 (Pig/HLJ/18), was isolated in primary porcine alveolar macrophages (PAMs) from a pig sample from an ASF outbreak farm. The isolate was characterized by using the haemadsorption (HAD) test, Western blotting and immunofluorescence, and electronic microscopy. Phylogenetic analysis of the viral p72 gene revealed that Pig/HLJ/18 belongs to Genotype II. Infectious titres of virus propagated in primary PAMs and pig marrow macrophages were as high as 10^7.2^ HAD_50_/ml. Specific-pathogen-free pigs intramuscularly inoculated with different virus dosages at 10^3.5^–10^6.5^ HAD_50_ showed acute disease with fever and haemorrhagic signs. The incubation periods were 3–5 days for virus-inoculated pigs and 9 days for contact pigs. All virus-inoculated pigs died between 6–9 days post-inoculation (p.i.), and the contact pigs died between 13–14 days post-contact (p.c.). Viremia started on day 2 p.i. in inoculated pigs and on day 9 p.c. in contact pigs. Viral genomic DNA started to be detected from oral and rectal swab samples on 2–5 days p.i. in virus-inoculated pigs, and 6–10 days p.c. in contact pigs. These results indicate that Pig/HLJ/18 is highly virulent and transmissible in domestic pigs. Our study demonstrates the threat of ASFV and emphasizes the need to control and eradicate ASF in China.

## Introduction

African swine fever (ASF) is one of the most serious viral diseases affecting pigs. It is listed as a “notifiable disease” by the World Organization for Animal Health (OIE), in part because of its high mortality rate. There is no effective vaccine to prevent ASF and it, therefore, poses a major threat to the pig industry and global food security [1]. ASF is caused by African swine fever virus (ASFV), which belongs to the genus *Asfivirus* within the *Asfarviridae* family [2,3]. Depending on the strain, ASFV has a large (170–193 kbp) double-stranded DNA genome containing 151–167 genes, which are involved in viral replication and assembly as well as in modulating host cellular functions and immune evasion [4].

ASFV was first identified in Kenya in the 1920s [5]. In the 1950s, ASFV rapidly spread throughout Europe, including Spain, Portugal, Italy, and France, and was eradicated from these countries, with the exception of Sardinia, by the mid-1990s [6]. However, the disease was introduced into Georgia in 2007, and then spread through Eastern Europe, including Russia, Belarus, Ukraine, Estonia, Lithuania, Latvia, Romania, Moldova, Czech Republic, and Poland [3]. The virus has continued to spread worldwide, and has now been reported in 37 countries or regions. In 2018, at least four countries, including Hungary, Bulgaria, Belgium, and China, reported their first ever ASF outbreaks to the OIE (http://www.oie.int/).

The first ASF case in China was reported on 3 August 2018. By January 19, 2019, at least 100 ASF cases had occurred in 23 provinces or regions across the country (http://www.oie.int/). The disease continues to spread in China, severely threatening the domestic pig population in China, which accounts for more than 50% of the pig population worldwide. To date, the diagnosis of ASF in China has been made on the basis of the detection of viral genes by means of real-time PCR and partial genome sequence analysis [7,8]. Accordingly, isolation and biological characterization of field viruses are urgently needed to assist in our understanding and control of the disease in China.

In this study, we isolated the first ASFV in China from a spleen sample from a pig on an ASF outbreak farm in Jiamusi city, Heilongjiang province, on 2 September 2018. The isolate was characterized by use of genotyping, the haemadsorption (HAD) test, Western blotting and immunofluorescence, and electronic microscopy. Primary porcine alveolar macrophage (PAM)-propagated virus was used to inoculate pigs at different doses to investigate incubation time, disease signs, viremia, contact transmission, pathogenicity, and viral loads in different organs and tissues.

## Materials and Methods

### Facility and ethics statements

All experiments with live ASF viruses were conducted within the enhanced biosafety level 3 (P3+) and level 4 (P4) facilities in the Harbin Veterinary Research Institute (HVRI) of the Chinese Academy of Agricultural Sciences (CAAS) approved by the Ministry of Agriculture and Rural Affairs. This study was carried out in strict accordance with the recommendations in the Guide for the Care and Use of Laboratory Animals of the Ministry of Science and Technology of the People’s Republic of China.

### Cell culture and virus isolation

Primary porcine alveolar macrophages (PAMs) were collected from 20–30-day-old SPF pigs, and the cells were maintained in 10% FBS RPMI 1640 medium (Thermo Scientific, USA) at 37°C with 5% CO_2_. PBMCs were prepared from EDTA-treated swine blood by using a pig PBMC isolation kit (TBD sciences, China). Porcine bone marrow (PBM) cells were collected as described previously [9].

The homogenate of the field pig spleen sample that was virus-positive by quantitative PCR (qPCR) and the haemadsorption (HAD) assay was used to inoculate PAMs for virus isolation. The cell supernatants were collected on day 4 post-inoculation. Virus in the supernatant was detected by using qPCR for viral gene copies and HAD assay for infectious virus particles.

### qPCR

ASFV genomic DNA was extracted by using GenElute™ Mammalian Genomic DNA Miniprep Kits (Sigma Aldrich, US) from cell supernatants, tissue homogenate, swabs, or EDTA-treated whole peripheral blood. qPCR was carried out on a QuantStudio 5 system (Applied Biosystems, USA) according to the OIE-recommended procedure described in King et al [10].

### HAD assay

The HAD assay was performed as described previously with minor modifications [9]. Primary PBM cells were seeded in 96-well plates. The samples were then added to the plates and titrated in triplicate using 10 × dilutions. The quantity of ASFV was determined by identification of characteristic rosette formation representing haemadsorption of erythrocytes around infected cells. HAD was observed for 7 days, and 50% HAD doses (HAD_50_) were calculated by using the method of Reed and Muench [11].

### Virus growth titration

Primary PAMs were infected at a multiplicity of infection (MOI) of 0.2 with ASFV isolate. Cell supernatants were collected at different times post-infection. Viral genomic DNA in cell supernatants was extracted and virus titres were determined by detecting viral p72 gene copy numbers by using qPCR.

### Western blot analysis

Primary PAMs were plated on 6-well plates and infected with ASFV at an MOI of 0.2. At 48 h post-infection, cells were collected, subjected to 12% SDS-PAGE under denaturing conditions, and transferred to polyvinylidene difluoride membrane. Anti-p30 mouse serum served as the primary antibody and horseradish peroxidase (HRP)-conjugated goat-anti-mouse IgG as the secondary antibody (Sigma, St. Louis, MO, USA). The bands were visualized with ECL Plus Western Blotting Detection Reagents (GE Healthcare Life Sciences, USA).

### Immunofluorescence assay

Primary PAMs were plated on 24-well plates and infected with ASFV at an MOI of 0.2. At 24 h post-infection, cells were fixed in 4% paraformaldehyde for 10 min at room temperature, permeabilized in 0.1% (w/v) Triton-100 for 10 min at room temperature, and then incubated with anti-p30 mouse serum for 1 h at room temperature. Cells were then washed 3 times with PBS and stained with FITC-conjugated goat anti-mouse antibody (Sigma, USA) for 45 min. Cell nuclei were stained with DAPI at room temperature for 1 h, and then washed 3 times with PBS. The FITC fluorescent signal was observed by using fluorescence microscopy (Axio Observer.Z1; Zeiss).

### Electron microscopy

Primary PAMs were seeded in 6-well plates and infected with ASFV at an MOI of 0.2. At 48 h post-infection, cell supernatants were collected and fixed with 2.5% glutaraldehyde (pH 7.2) for negative staining. An aliquot of 20 μL of sample was applied to a carbon-coated grid that had been glow discharged. The grid was negatively stained with 2% phosphotungstic acid. In addition, ASFV-infected cells were washed with PBS, fixed with 2.5% glutaraldehyde (pH 7.2) at 4°C overnight, post-fixed with 1% OsO4 (pH 7.4) at 4°C for 2 h, dehydrated in stepwise acetone at 4°C, and embedded in 812 Epon resin. Thin sections were stained with 1% uranyl acetate (pH 6.5) and 1% lead citrate (pH 7.2). The grid was examined in an H-7650 (Hitachi, Tokyo, Japan) operated at 80 kV.

### Animal experiments

Animal experiments were performed within the animal biosafety level 4 facilities at HVRI following a protocol approved by the Animal Ethics Committee of HVRI of CAAS and the Animal Ethics Committee of Heilongjiang Province, China.

Seven-week-old SPF Large White and Landrace-crossed pigs were obtained from the Laboratory Animal Center of HVRI, and divided into 4 groups. One pig was intramuscularly inoculated with virus at a dose of 10^6.5^ HAD_50_, two pigs were inoculated with 10^5.5^ HAD_50_, and two groups of three pigs were inoculated with 10^4.5^ and 10^3.5^ HAD_50_, respectively. The pigs inoculated with 10^4.5^ and 10^3.5^ HAD_50_ were also co-housed with one naïve pig, beginning on the first day of infection, respectively, to evaluate ASFV contact transmission. The pigs were monitored daily for temperature and clinical signs (anorexia, depression, fever, purple skin discoloration, staggering gait, diarrhea, and cough). Blood and oral and rectal swabs were collected every day for virus detection. Necropsy was immediately performed if the animal died during the day, or the next morning if the animal died at night, and tissues were collected for further analysis.

## Results

### Isolation and characterization of ASFV in vitro

**Figure 1.**
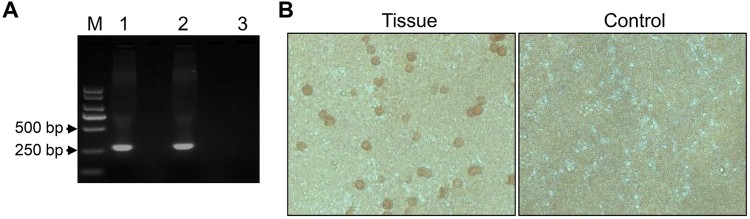
Detection of ASFV in a field sample of spleen tissue. (A) PCR detection of spleen DNA from the suspected pig. Lane 1, sample; lane 2, positive control plasmid; lane 3, negative control. (B) HAD assay of the spleen homogenate. The 10-times dilution of the supernatant of the homogenate was inoculated into pig PBMCs with 1% pig blood cells. HAD was observed for 7 days.

We performed PCR targeting the sequence of the p72 gene to confirm that the field spleen sample was positive for ASFV as recommended by the OIE [12], and a positive 257-bp product was obtained (Figure 1(A)). On day 2 post-inoculation (p.i.) with the supernatant of the spleen homogenate, primary PAMs showed clear HAD, even in the wells inoculated with 100-fold diluted sample (Figure 1(B)). These results confirmed that the spleen homogenate contained infectious ASFV.

**Figure 2.**
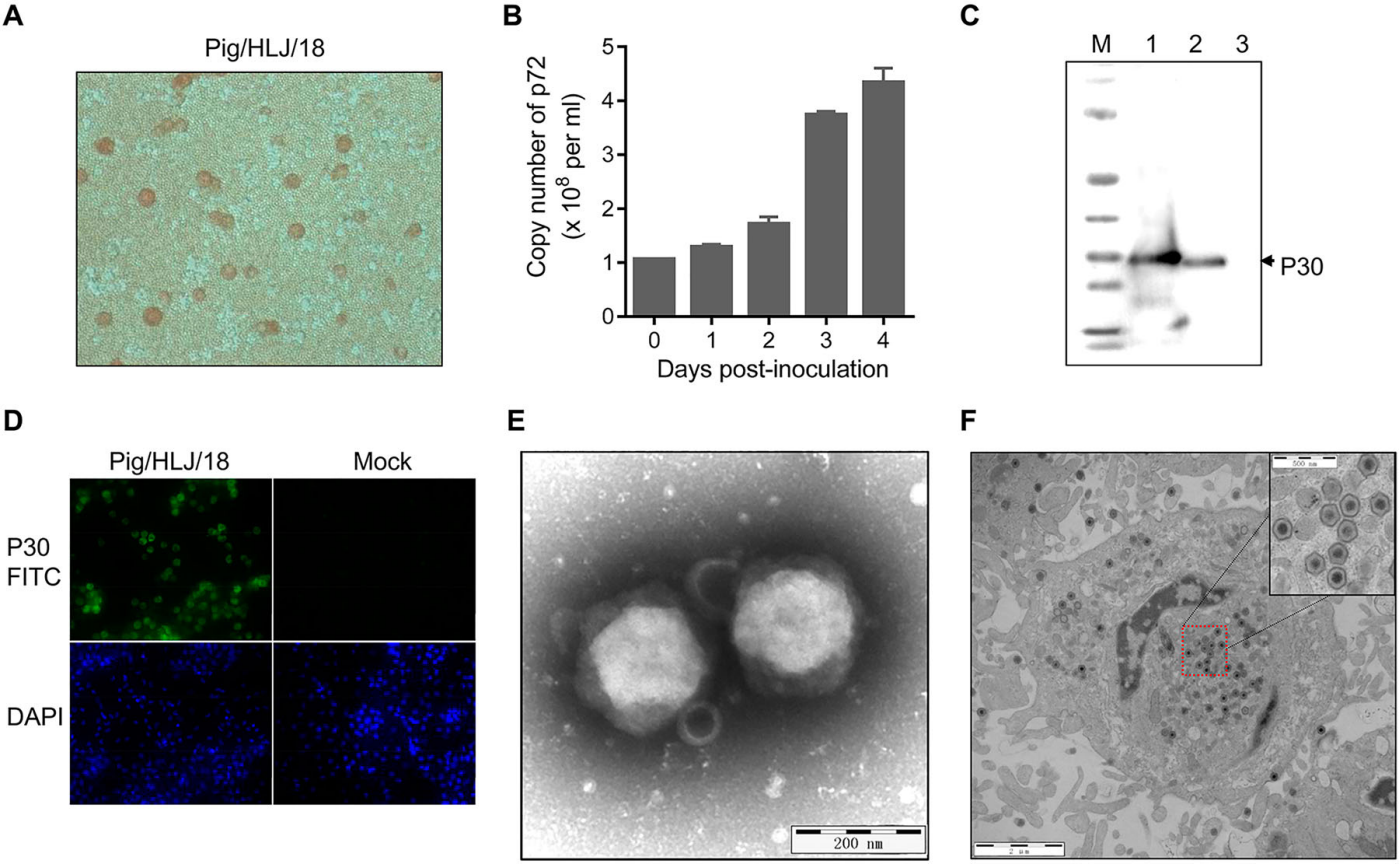
Viral growth in primary PAMs. Primary PAMs were infected with Pig/HLJ/18 at an MOI of 0.2, and the supernatants were collected for infectious virus particle detection and quantification of viral p72 gene copies by using HAD (A) and qPCR (B) assays, respectively. The expression of viral p30 in the infected primary PAMs was detected by Western blotting of the lysate of cells infected with Pig/HLJ/18 (C, line 1) [cells transfected with pCAGG-p30 (C, line 2)] and by use of an immunofluorescence assay with cells infected with Pig/HLJ/18 (D). Electron microscopic observation of ASFV particles in cell culture supernatants by negative staining (E), and in infected cells by ultrathin-sectioning (F) at 48 h post-infection.

To isolate the virus from the spleen homogenate, we inoculated it into primary PAMs and collected cell supernatants as the first passage stock on day 4 p.i.. The HAD assay was ASFV-positive for the virus stock (Figure 2(A)). The titre for the first passage stock was 10^7.2^ HAD_50_/ml. To assess the viral growth dynamics, primary PAMs were infected at a multiplicity of infection (MOI) of 0.2, and the cell supernatants were collected at different times post-infection for viral genome quantification by qPCR targeting the p72 gene [10]. The viral genome copy numbers in the cell supernatants reached 4.3 × 10^8^/ml on day 4 p.i. (Figure 2(B)). The early viral structural protein p30, which is encoded by the CP204L gene, was detected as early as 2–4 h p.i. and throughout the viral replication cycle [13]. Therefore, we examined the expression of p30 in primary PAMs infected with the virus stock and detected it by Western blotting (Figure 2(C)) and the use of an indirect immunofluorescence assay (Figure 2(D)). The typical morphology of ASFV particles in the cell cultural supernatant (Figure 2(E)) and cytoplasm of primary PAMs (Figure 2(F)) was also observed electron microscopically. These results demonstrate that ASFV was successfully isolated from the field outbreak sample. This virus isolate was designated as Pig/Heilongjiang/2018, abbreviated as Pig/HLJ/18.

### Sequence analysis of the ASFV isolate

**Figure 3.**
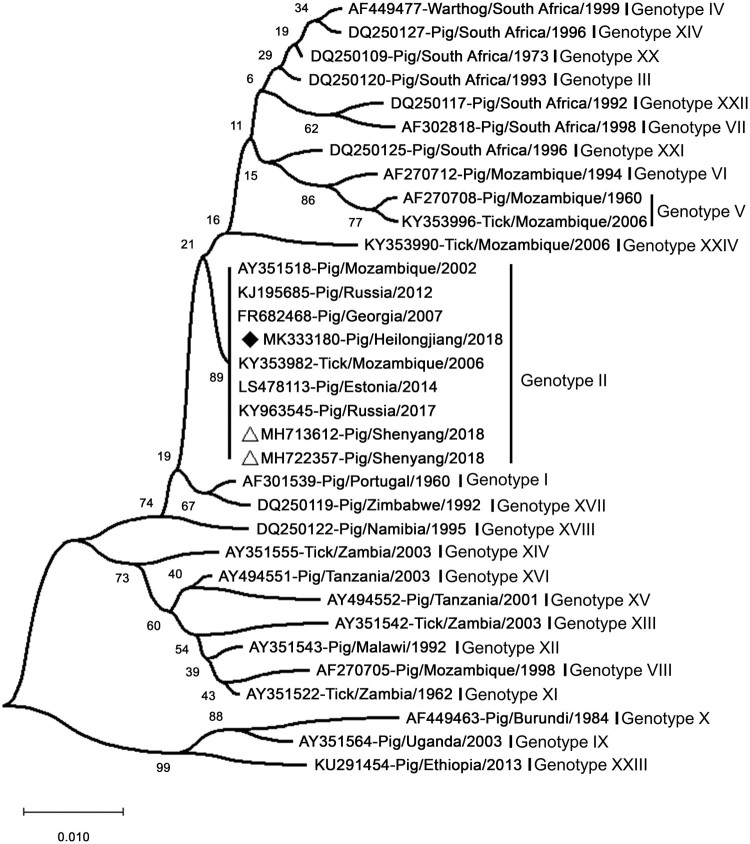
Phylogenetic analysis of Pig/HLJ/18 based on its partial p72 gene. The sequences of the p72 genes of representative ASFVs were downloaded from the NCBI database. The neighbor-joining method was used to construct phylogenetic trees using MEGA X software (https://www.megasoftware.net/). Numbers along branches indicate bootstrap values >70% (1,000 replicates). The black diamond indicates the ASFV isolate from this study. The white triangle marks the ASFV sequences from the first case in China. Scale bars indicate nucleotide substitutions per site.

Sequence analysis of p72 suggested that Pig/HLJ/18 shares high identity with Georgia/2007/1 and the virus ASFV-SY-18 that caused the first ASF case in Shenyang in China on August 3 [7,8]. Phylogenetic analysis based on partial p72 genes showed that Pig/HLJ/18 belongs to Genotype II and is genetically close to ASFVs prevalent in Eastern Europe (Figure 3). However, the full genome sequence analysis recently reported by Wen et al. [14] showed that Pig/HLJ/18 has the highest similarity with the sequence of an ASFV isolated in Poland in 2017 [PoL/2017 (GenBank: MG939588.1)], and a comparison with the sequences of Georgia/2007/1 (GenBank: FR682468.1) and ASFV-SY-18 (GenBank: MH766894.1), revealed that nucleotide insertions, deletions, and mutations had occurred at multiple positions in the genomes of the PoL/2017 and Pig/HLJ/18 viruses.

### The virulence of Pig/HLJ/18 in domestic pigs

Different pathotypes of ASFVs have been detected in nature [15,16]. There were 87 pigs on the farm where Pig/HLJ/18 was isolated; 39 pigs were sick and 12 of them died [14]. Therefore, from an epidemiological standpoint, it is difficult to assess the lethality of Pig/HLJ/18 to pigs. To characterize the pathogenicity of Pig/HLJ/18, nine specific-pathogen-free (SPF) pigs were infected. One pig was intramuscularly inoculated with virus at a dose of 10^6.5^ HAD_50_, two pigs were inoculated with 10^5.5^ HAD_50_, and two groups of three pigs were inoculated with 10^4.5^ and 10^3.5^ HAD_50_, respectively. The pigs inoculated with 10^4.5^ and 10^3.5^ HAD_50_ were also co-housed with one naïve pig, beginning on the first day of infection, respectively, to evaluate ASFV contact transmission. The disease signs, including fever (over 41°C), loss of appetite, depression, lethargy, redness of the skin, cyanosis on the edges of the ears and ends of the tail and legs, respiratory distress, and vomiting, started 3–5 days p.i. in all of the virus-inoculated pigs. The pigs that received 10^6.5^ HAD_50_ and 10^5.5^ HAD_50_ of the virus died between 6–7 days p.i.. Six animals that received the two lower doses of 10^4.5^ and 10^3.5^ HAD_50_ of the virus died between 6–9 days p.i. (Table 1). These results demonstrate that Pig/HLJ/18 is highly lethal to domestic pigs with a 50% pig lethality dose (PLD_50_) of less than 10^3.0^ HAD_50_.

**Table 1.** Replication and virulence of the African swine fever virus Pig/HLJ/18 in specific-pathogen-free pigs.

Virus inoculation dose (HAD_50_)	The earliest time viral genomic DNA was detected in blood or swabs by qPCR [day post inoculation or contact (dpi or dpc)]			Death time (dpi or dpc)	Highest titre of virus in organs of dead pigs (Copy number of the viral p72 gene per gram of tissue)				
	Blood	Oral swab	Rectal swab		Liver	Spleen	Heart	Colon	Submaxillary lymph node
10^6.5^	2	2	3	7	2.0 × 10^10^	8.0 × 10^9^	2.0 × 10^9^	1.0 × 10^10^	4.0 × 10^9^
10^5.5^	2	4	4	6–7	5.9 × 10^9^	1.8 × 10^10^	8.0 × 10^8^	1.1 × 10^9^	4.8 × 10^9^
10^4.5^	3	4	5	7–9	2.5 × 10^10^	7.9 × 10^10^	3.6 × 10^9^	4.9 × 10^8^	2.0 × 10^9^
10^3.5^	2	4	5	6–8	1.7 × 10^9^	3.8 × 10^9^	2.7 × 10^8^	9.2 × 10^7^	5.9 × 10^9^
Contact pig 1^a^	9	6	10	13	5.2 × 10^7^	2.5 × 10^8^	8.0 × 10^6^	4.9 × 10^6^	1.9 × 10^8^
Contact pig 2^a^	9	6	10	14	2.0 × 10^8^	2.0 × 10^8^	1.0 × 10^7^	7.0 × 10^5^	5.0 × 10^6^

Contact pig 1 is the pig that co-housed with the pigs that received 10^4.5^ HAD_50_ of Pig/HLJ/18 virus, and contact pig 2 is the pig that co-housed with the pigs that received 10^3.5^ HAD_50_ of Pig/HLJ/18 virus.

**Figure 4.**
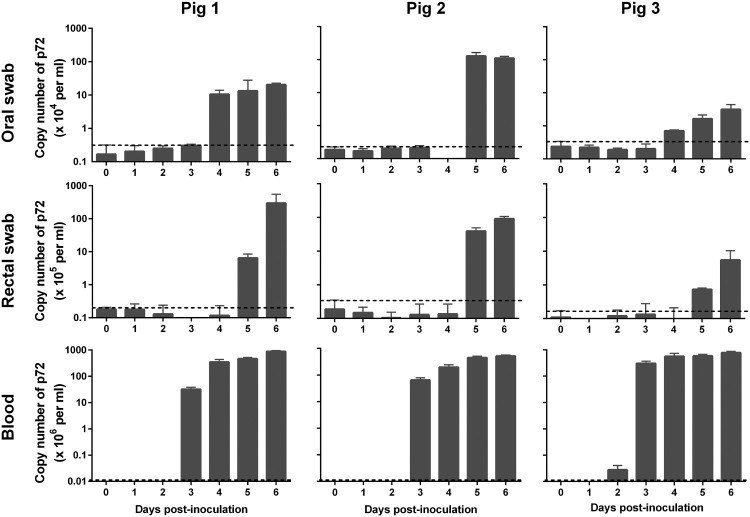
Viral genomic copies in samples collected from Pig/HLJ/18 infected pigs. Blood, as well as oral and rectal swab samples, were collected at the indicated time points from three pigs inoculated with 10^3.5^ HAD_50_ of Pig/HLJ/18. Viral p72 gene copies were quantified by using the qPCR assay. The dashed black lines in these panels indicate the limit of detection compared with the data of samples collected at day 0 post-inoculation.

**Figure 5.**
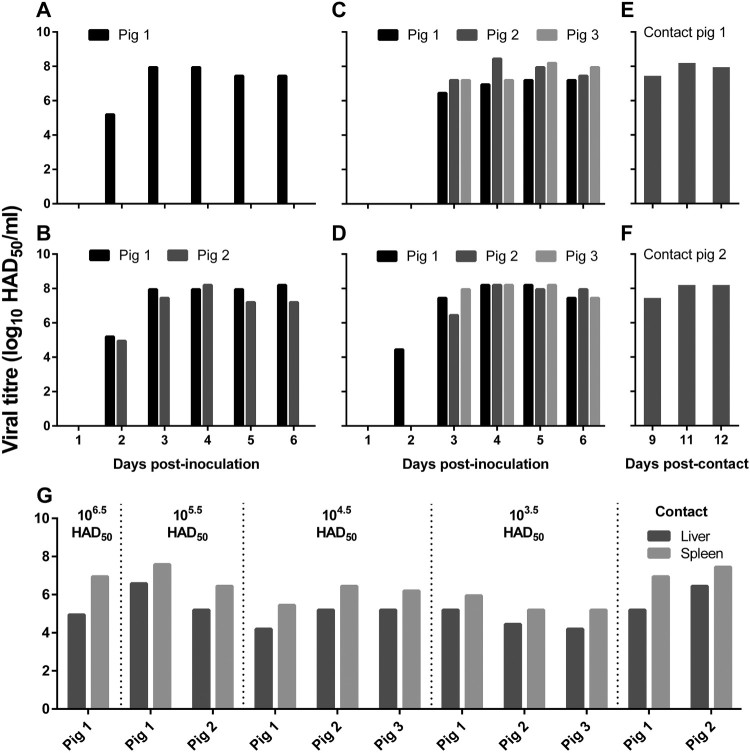
Viral titres in the blood and organs of pigs tested by using the HAD assay. Blood was collected from pigs that were inoculated with different doses of ASFV Pig/HLJ/18 (A─D) or from pigs that contacted with pigs that were inoculated with ASFV Pig/HLJ/18 (E─F) at the indicated time points. (A) 10^6.5^ HAD_50_-inoculated pig; (B) 10^5.5^ HAD_50_-inoculated pigs; (C) 10^4.5^ HAD_50_-inoculated pigs; (D) 10^3.5^ HAD_50_-inoculated pigs; (E) Pig co-housed with pigs inoculated with 10^4.5^ HAD_50_ of ASFV Pig/HLJ/18; (F) Pig co-housed with pigs inoculated with 10^3.5^ HAD_50_ of ASFV Pig/HLJ/18. (G) Viral titres in the liver and spleen from virus-inoculated pigs and contact pigs. Samples were collected during necropsy and the virus inoculation dose for each pig group is shown at the top of the panel.

Blood, oral swabs, and rectal swabs were collected from virus-inoculated pigs and assessed by qPCR for the p72 gene. Viral genomic DNA started to be detectable from blood on 2–3 days p.i. (Table 1), from the oral swabs on 2–4 days p.i. and from the rectal swabs on 3–5 days p.i. (Table 1 and Figure 4). Viral genomic DNA detection from the pigs inoculated with the 10^6.5^ HAD_50_ dose occurred two days earlier than that from the pigs inoculated with the 10^3.5^ HAD_50_ dose (Table 1 and Figure 4). The blood samples collected from 1 to 6 days p.i. were also examined in HAD assays and the results were consistent with qPCR data (Figure 5(A-D)).

### Transmission of Pig/HLJ/18 in pigs

To evaluate the transmissibility of the ASFV isolate Pig/HLJ/18 in pigs, two naive pigs were, respectively, housed with pigs inoculated with 10^4.5^ HAD_50_ or 10^3.5^ HAD_50_ of Pig/HLJ/18 from the first day of infection. Blood samples, as well as oral and rectal swabs, were collected from the two contact pigs for virus detection by qPCR, and pigs were observed for disease signs every day p.c..

**Figure 6.**
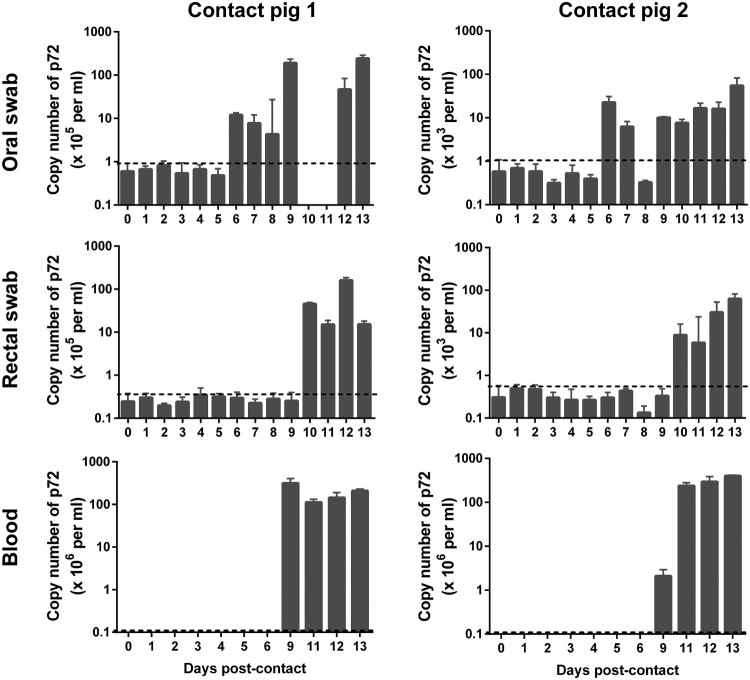
Viral genomic copies in samples from contact pigs. Blood, as well as oral and rectal swab samples were collected from two contact pigs, which were housed with pigs inoculated with 10^4.5^ HAD_50_ (contact pig 1) and 10^3.5^ HAD_50_ (contact pig 2), respectively, at different timepoints. The dashed black lines in these panels indicate the limit of detection compared with the data of samples collected at day 0 post-inoculation.

As shown in Figure 6, viral DNA started to be detected from the oral swabs on day 6 p.c., from the rectal swabs on day 10 p.c., and from blood samples on day 9 p.c.. Blood samples were also tested by using the HAD assay (Figure 5(E-F)), and the results were consistent with the qPCR data. Compared to oral samples, blood and rectal swab samples were delayed by about 3–4 days in terms of testing positive for viral DNA, which indicates that the two contact pigs were infected via the oral or nasopharyngeal route at around 5–6 days p.c..

The contact pigs did not show any signs of disease during the first 8 days p.c., but started to show signs of reduced appetite, fever, depression, lethargy, and redness of the skin on day 9 p.c.. The two contact pigs died on day 13 and day 14 p.c., respectively.

### Lesions and virus replication in organs

**Figure 7.**
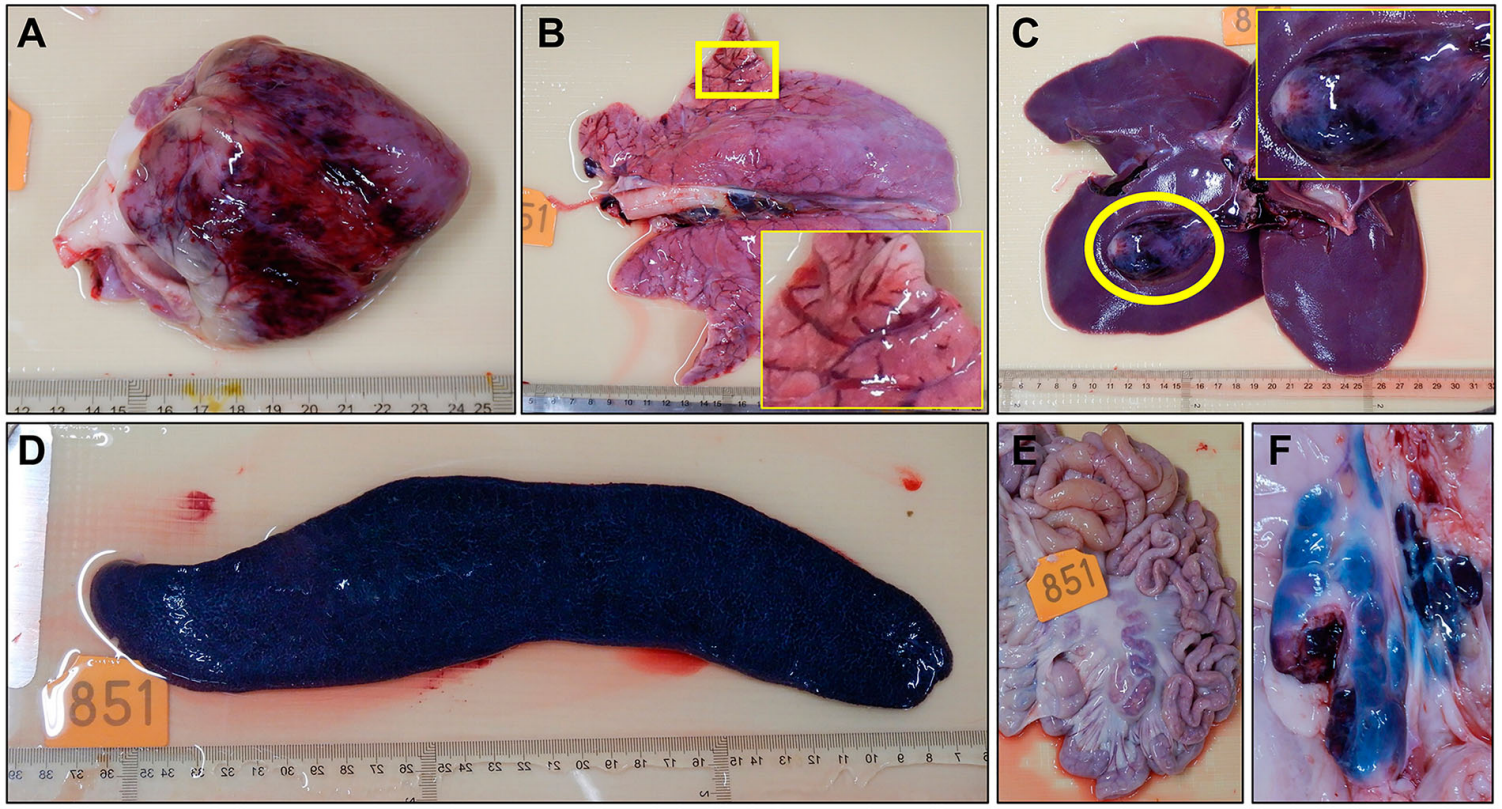
Gross lesions in pigs that died as a result of infection with Pig/HLJ/18. The gross lesions of all pigs are summarized and shown in Supplementary Table. The lesions of the heart, lung, gall bladder, spleen, mesenteric lymph nodes, and gastro-hepatic lymph nodes of the pig that was inoculated with 10^6.5^ HAD_50_ of the virus are shown here. **A**. Severe and diffuse haemorrhage of the epicardium in the heart. **B**. Mild interstitial oedema of the lung; the insert is a higher magnification of the framed area. **C**. Severe haemorrhage and oedema of the gallbladder wall and liver; the insert is a higher magnification of the circled area. **D**. Severe, enlarged, friable, and dark black appearance of the spleen. **E**. Mesenteric lymph nodes, showing mildly enlarged and red-black appearance. **F**. Gastro-hepatic lymph nodes appear very enlarged and haemorrhagic.

Necropsy was performed for all eleven pigs that died. The gross lesions of the pigs are summarized and shown in Supplementary Table. Cyanosis of ears was observed in six pigs. Severe epicardial, subendocardial, and myocardial haemorrhage were only observed in the pig inoculated with the 10^6.5^ HAD_50_ of virus (Supplementary Table, Figure 7(A)). Haemorrhage of lymph nodes was observed in all 11 pigs, splenomegaly was observed in nine of the 11 pigs, and haemorrhage of the kidney was observed in eight of the 11 pigs (Supplementary Table). Other lesions, including hydropericardium, hemorrhage/oedema of the gall bladder, pulmonary oedema, haemorrhage on the serosa and mucosa of the large intestines, haemorrhage of the urinary bladder, and gastric ulcer/haemorrhage were observed in 2–6 pigs (Supplementary Table). The lesions of the heart, lung, gall bladder, spleen, mesenteric lymph nodes, and gastro-hepatic lymph nodes of the pig that was inoculated with 10^6.5^ HAD_50_ of the virus are shown in Figure 7.

Tissue samples including heart, liver, spleen, colon, and submaxillary lymph node were collected from all of the pigs for qPCR analysis. Viral genomic DNA was detected from all tested tissues, and the liver, spleen, and the lymph node generally showed higher viral loads than the other tissues (Table 1). The presence of infectious virus in the liver and spleen was also confirmed by use of the HAD assay (Figure 5(G)).

## Discussion

In this study, we successfully isolated the first ASFV from a field outbreak sample in China. The isolate, Pig/HLJ/18, was characterized by use of the HAD assay, electron microscopic observation, and Western blotting and immunofluorescence assays. The virus belongs to Genotype II and is genetically close to ASFVs prevalent in Eastern Europe; it replicated efficiently in primary PAMs and its viral titre reached 10^7.2^ HAD_50_/ml. Animal studies demonstrated that the Pig/HLJ/18 virus replicates systemically in pigs, is highly virulent in pigs, and is efficiently transmissible among pigs.

The incubation period of ASF varies among viral strains and animal species, ranging from 3–19 days according to previous studies [17–21]. In our study, pigs inoculated with different dosages of Pig/HLJ/18 started to show early disease signs by 3–5 days p.i., and all of the animals died between 6–9 days p.i. Two contact pigs showed the onset of early disease signs on day 9 p.c., and died on days 13 and 14 p.c., respectively, indicating that the Pig/HLJ/18 virus causes very acute disease in SPF pigs; its incubation period was 3–5 days in virus-inoculated pigs and about 9 days in the contact pigs. Of note, the disease signs and necropsy changes caused by ASFV are similar with those caused by other pig acute diseases, such as classical swine fever and highly pathogenic porcine reproductive and respiratory syndrome [3,12,21–23]; therefore, virological tests are indispensable for the diagnosis of ASF.

Transmission of ASFVs among pigs occurs through multiple routes. Previous studies suggest that the ASFVs can transmit from infected pigs to naïve pigs through virus-contaminated feces and air [18,24–26]; virus DNA detection in air samples was significantly associated with the detection of virus DNA in feces [18,27]. Bleeding from the introitus was not observed in any virus-inoculated pigs or contact pigs in our study, indicating the contact pigs may have been infected by sharing feed or water with the virus-inoculated pigs or via contact with the feces of the virus-inoculated pigs. However, our study and the studies by others [21,25] indicate that blood contains very high virus titres, and therefore blood may be an important source for transmitting viruses to naïve contact animals. First, studies have reported that pigs (9.7%–36.1%) bleed from an introitus, such as the mouth, nose, anus, or vagina, at the later stage of acute ASFV infection [28,29], and naïve pigs that come into direct contact with the blood or blood-contaminated environment will certainly be infected by the ASFV. Second, pig blood is commonly collected from pig slaughterhouses and then used as feed for pigs and other animals as an important protein source. If blood for feed is collected from ASFV virus-infected pigs and is not properly inactivated, it would be an important source for ASFV transmission.

The virulence of ASFV is determined by both virus and host. The ASFV usually causes a persistent but asymptomatic infection in its natural hosts, which include warthogs, bush pigs, and soft ticks [3,30,31]. In domestic pigs and Eurasian wild boars, however, disease signs of ASF vary from peracute to chronic disease, and apparently healthy virus carriers are asymptomatic [28,32]. The virulence of different ASFVs in the same host may vary among different strains. To our knowledge, Pig/HLJ/18 is the only ASF virus isolated and tested in pigs in China to date. The Pig/HLJ/18-inoculated pigs died within nine days of inoculation and the contact pigs died within two weeks of contact with inoculated pigs, which demonstrates that a highly virulent ASFV has entered the pig population in China. Although tremendous efforts have been made to control each outbreak, ASF is continuously spreading. As of January 19, 2019, ASF outbreaks have occurred in pigs in over 23 provinces or regions in China (http://www.oie.int/). It is still not known if a single virus invaded in China and caused all of these outbreaks or if different ASFVs are responsible for the outbreaks. More viruses need to be isolated and analyzed to fully understand the spread of the disease and to develop an effective control strategy.

## Supplementary Material

Supplemental Material
