# Supplementary material for: Replication and virulence in pigs of the first African swine fever virus isolated in China
Source: Emerg Microbes Infect. 2019 Mar 22;8(1):438–47. doi: 10.1080/22221751.2019.1590128 (PMC6455124; doi:10.1080/22221751.2019.1590128)
Supplement: Supplemental Material [file TEMI_A_1590128_SM3478.docx]

**Appendices:**

**Supplementary Table. Gross lesions of pigs infected or in contact with the African swine fever virus (ASFV) Pig/HLJ/18.**

| Gross lesion | Animal treatment | | | | | | | | | | |
| --- | --- | --- | --- | --- | --- | --- | --- | --- | --- | --- | --- |
|  | Pigs inoculated with different doses of ASFV pig/HLJ/18 (HAD_50_)^a^ | | | | | | | | | Contact pigs^b^ | |
|  | 10^6.5^ | 10^5.5^ | | 10^4.5^ | | | 10^3.5^ | | |  |  |
|  | Pig 1 | Pig 1 | Pig 2 | Pig 1 | Pig 2 | Pig 3 | Pig 1 | Pig 2 | Pig 3 | Pig 1 | Pig 2 |
| Cyanosis of the ears | ++ | + | - | + | - | - | + | - | + | - | + |
| Hydropericardium | +++ | ++ | - | - | - | + | - | - | - | - | + |
| Haemorrhage of the heart | +++ | - | - | - | - | - | - | - | - | - | - |
| Haemorrhage/oedema of the gall bladder | +++ | - | - | - | + | - | - | - | - | - | - |
| Haemorrhage of the intestines^c^ | +++ | ++ | - | - | - | ++ | - | - | - | - | - |
| Pulmonary oedema | ++ | + | + | - | + | + | - | - | - | - | - |
| Haemorrhage of the kidney | + | ++ | + | ++ | + | + | + | - | + | - | - |
| Splenomegaly | +++ | + | + | - | ++ | - | + | + | + | + | + |
| Haemorrhage of the lymph nodes^d^ | +++ | ++ | + | + | ++ | + | + | ++ | ++ | + | ++ |
| Haemorrhage of the urinary bladder | - | ++ | - | ++ | + | - | + | ++ | ++ | - | - |
| Gastric ulcer/ haemorrhage | - | +++ | - | - | +++ | - | + | + | - | - | - |

1. Lesion scale: -, No obvious change; +, Mild; ++, Moderate; +++, Severe.
2. Contact pig 1 is the pig that was co-housed with the pigs that received 10^4.5^ HAD_50_ of Pig/HLJ/18 virus; contact pig 2 is the pig that was co-housed with the pigs that received 10^3.5^ HAD_50_ of Pig/HLJ/18 virus.
3. Lesions were mainly observed in the cecum, colon, and rectum of the pigs.
4. Lesions were mainly observed in the submaxillary lymph nodes, mesenteric lymph nodes, and gastro-hepatic lymph nodes of the pigs.
